# The Papers at the Recent Meeting of the Medical Association of Georgia

**Published:** 1908-05

**Authors:** 


### THE PAPERS AT THE RECENT MEETING OF THE MEDICAL ASSOCIATION OF GEORGIA.

On looking back a decade or so to the meetings of this Association, the impartial observer is happily impressed with the change which has taken place in the general character of the contributions to the scientific aspect of these gatherings. Then though there were some excellent discourses, one was not yet usually exactly spell-bound by what one listened to. Now there is something to be proud of. There are clear traces of originality, thought, and earnestness in no inconsiderable proportion of the papers presented. It would be foolish to plume ourselves on present achievements for there is much, very much, still to criticise in the medicine side of life in Georgia. But movement is clearly forward and upwards, and hope is well grounded. In the mean time there are a few men among us who are doing first-class work as investigators, and a considerable number who are building up a high reputation for themselves and their state. Excelsior! S.

